# Severe imported falciparum malaria among adults requiring intensive care: a retrospective study at the hospital for tropical diseases, London

**DOI:** 10.1186/1471-2334-13-118

**Published:** 2013-03-05

**Authors:** Michael E Marks, Margaret Armstrong, Muhiddin M Suvari, Steve Batson, J M Christopher Whitty, Peter L Chiodini, Geoff Bellinghan, Justin F Doherty

**Affiliations:** 1 The Hospital for Tropical Diseases, Mortimer Market Centre, Capper Street, WC1E 6JB, London, UK; 2 Department of Clinical Parasitology, Hospital for Tropical Diseases, Mortimer Market Centre, Capper Street, WC1E 6JB, London, UK; 3 Intensive Care Unit, University College London Hospitals, Euston Road, NW1, London, UK

## Abstract

**Background:**

Malaria is the commonest imported infection in the UK. Malaria requiring ICU admission has a reported mortality of up to 25%. The relationship between ethnicity, immunity, and risk of malaria is complex. The Malaria Score for Adults (MSA) and Coma Acidosis Malaria (CAM) score have recently been proposed to risk stratify patients with malaria.

**Methods:**

Retrospective study of patients with WHO severe falciparum malaria admitted to ICU at the Hospital for Tropical Diseases, London, UK. The relationship between clinical variables and risk of death or a prolonged ICU stay were examined with logistic regression. The predictive value of the MSA and CAM score were calculated.

**Results:**

124 patients were included. Cerebral malaria and acute kidney injury occurred earlier (median day 1) than acute respiratory distress syndrome (median day 3). Six patients had community acquired bacterial co-infection. Eight patients were co-infected with HIV, five of whom were newly diagnosed. The positive predictive value of a CAM score ≥2 or an MSA ≥5 for death were 12% and 22% respectively. Five patients died. No variable was significantly associated with risk of death. There were no significant differences between individuals raised in endemic countries compared to non-endemic countries.

**Conclusions:**

Mortality in patients managed in a specialist centre was low. Patients who died succumbed to complications associated with a prolonged stay on ICU rather than malaria *per se*. The clinical usefulness of the MSA and CAM score was limited. Co-infection with HIV was relatively common but compared to studies in children, bacteraemia was uncommon. The relationship between ethnicity and immunity to severe disease is complex.

## Background

Approximately 1700 cases of imported malaria are seen each year in the United Kingdom, of which *P*. *falciparum* is both the most common and the most severe [1], and it remains the most significant imported parasitic infection in North America, Europe and Australasia [2-4]. While the number of deaths from imported malaria is low, it has the potential to cause significant morbidity. The case fatality rate for *P*. *falciparum* in the UK is less than 1% but in previous series the mortality rate for those patients requiring admission to an intensive care unit (ICU) as a result of malaria has varied from 7% to 25% [5-11]. Most data on severe malaria come from endemic countries. Severe imported malaria seen in non-endemic countries is a very different clinical entity, particularly in terms of the resources available to care for critically ill patients, median age at presentation (mainly children in Africa and young adults in Asian series) and the likely effect of partial immunity from previous infection on disease progression in endemic countries.

Identifying those at high risk of death is important for subsequent treatment decisions. The Glasgow Coma Score (GCS) is widely accepted as a tool for identifying patients most at risk of a poor outcome and cerebral malaria in a non-endemic setting is usually defined for practical purposes as a GCS <11. Recently two scoring systems – Malaria Score in Adults (MSA) [12] and the Coma Acidosis Malaria (CAM) Score [13] have been suggested as additional systems to identify those patients at higher risk of death. However, neither of these has previously been validated among patients with imported malaria.

Earlier reports of severe imported malaria have shown that cerebral malaria, adult respiratory distress syndrome (ARDS) and acute kidney injury (AKI) are the most common complications of malaria requiring admission to ICU [5-11]. The inclusion criteria for these studies have varied; some included patients with non-severe malaria admitted to ICU while others included all patients with malaria who met at least one of the World Health Organisation’s (WHO) criteria for severe disease [14] regardless of whether or not they were admitted to ICU. Definitions of hyperparasitaemia also varied with 2%, 4% and 5% taken as cut-off values in different papers. Only two papers [5,6] have included data on the rates of bacterial co-infection. None of these earlier studies have included patients treated with Artemisinin-based therapy and in only one study [11] were exchange transfusions used. Data on any long term sequelae following severe imported malaria are also scarce.

The concept of immunity to malaria is widely accepted but poorly understood. In areas of high endemicity, children gradually acquire immunity to the disease as a result of repeated exposure to infection, such that by the age of approximately five years severe malaria is unlikely to occur. Should such a child then move away from an endemic area, this immunity is thought to be lost relatively quickly and, within a matter of months or years, the child would again be at risk of severe disease. Unfortunately, no laboratory marker of “immunity” to malaria has been identified. The length of time that an individual has lived in a malaria-endemic area is often used as a proxy for immunity. Conversely, people who were born and raised in parts of the world where malaria transmission does not occur are widely assumed to be “naïve” and therefore at higher risk of severe disease. We aimed to examine whether any previous exposure to malaria would have an appreciable effect on the manifestations of severe malaria by stratifying patients according to their perceived “immunity” [15].

The inter-relationship between malaria and HIV infection has been studied almost exclusively in endemic areas. Co-infection with malaria has been shown to have a detrimental effect on HIV viral load and to result in more frequent and more severe disease [16]. No data have been reported concerning the prevalence of HIV infection among patients with severe imported malaria.

This study therefore set out to examine the main questions around management of severe malaria in high-resource non-endemic setting.

## Methods

The Hospital for Tropical Diseases (HTD) in London is part of University College London Hospitals and serves as a tertiary referral centre for patients with imported tropical diseases, including malaria. The hospital sees approximately 75 patients with falciparum each year, of which about 10% are sufficiently unwell to require admission to ICU.

### Study population

A retrospective study was conducted on all adult patients (>18 years) seen at HTD/UCLH between 1994 and 2010. During this period a total of 1616 patients with falciparum malaria were seen, of whom 165 were sufficiently unwell to require admission to intensive care. Patients were excluded if they did not meet at least one of the WHO criteria for severe disease (parasitaemia >5%, ARDS [bilateral pulmonary infiltrates and PaO_2_:FiO_2_ ratio of <26.7, not attributed to left ventricular dysfunction in the opinion of the ICU clinician], impaired consciousness [seizures or GCS <11], multiple convulsions, shock [systolic BP <80 mmHg despite adequate filling], abnormal bleeding or coagulopathy [unexplained clinically significant bleeding or laboratory evidence of DIC), macroscopic haemoglobinuria, AKI [creatinine >265 μmol/L], jaundice [bilirubin >50 μmol/L], hyperlactaemia [arterial lactate >5 mmol/L], acidosis [pH <7.35 or serum bicarbonate < 15 mmol/L], hypoglycaemia [blood glucose <2.2 mmol/L], severe anaemia [haemoglobin <5 g/dL]) [12]. The diagnosis of malaria was made by microscopic examination of a blood film. Microscopy was used to quantify parasitaemia, the presence of schizonts and co-infections with other forms of *Plasmodia* (*vivax*, *ovale*, *malariae*). Most were referred to HTD from other hospitals where they had already received treatment for malaria and, if there was no evidence of asexual parasitaemia at the time of admission to HTD/UCLH ICU, they were excluded. Of 165 patients admitted to ICU, 32 had notes which could not be found, eight no longer had asexual parasites on their blood film and one did not fulfil any of the WHO criteria for severe disease, leaving a total study population of 124.

### Data collection

Demographic, travel, clinical, laboratory and modalities of treatment data were collected from clinical notes and laboratory databases using a standard case report form. Complications of malaria were defined according to WHO guidelines on severe malaria [14]. Blood cultures that were positive within 48 hours of admission were taken to represent community-acquired infection; positive cultures after this time were defined as nosocomial. For the purposes of analyses, patients were classified as “malaria-naïve’ if they had been born and raised outside a malaria-endemic area. Patients who were born in an endemic area but who had moved away a minimum of two years earlier were classified as having “little” immunity to malaria. Patients who were born and still resident in an endemic area at the time of admission were assumed to have “some” immunity to malaria.

### Management

All patients were managed according to UK guidelines [17]. Until 2007, these recommended a loading dose of quinine (20 mg/kg) given intravenously over 4 hours, followed by 10 mg/kg until such time as a blood film was negative for asexual parasites. Exchange transfusion was considered for patients with a parasitaemia greater than 20% or those who had a parasitaemia greater than 10% with evidence of end-organ dysfunction. After 2007, intra-venous artesunate replaced quinine as first-line treatment for patients with a parasitaemia greater than 10% at HTD, depending on drug availability. All patients received a second anti-malarial agent, most commonly sulfadoxine/pyrimethamine. Decisions concerning organ support and adjunctive therapy were made exclusively by ICU staff.

### Statistical analyses

Continuous variables were described with mean and standard deviation (SD) or with median and interquartile range (IQR) as appropriate. Categorical data were described with numbers and percent. “Severe ICU malaria” was defined as death or an ICU stay greater than seven days. The patients’ MSA and CAM scores on admission to hospital were calculated as previously described [12,13]. The relationship between the MSA and CAM scores and risk of death and severe ICU malaria were compared using chi-square. Logistic regression was used to examine variables, including in the model the presence or absence of complications, age, gender and immune status, to determine factors associated with either death or severe ICU malaria. All analyses were carried out using Stata 10 (Statacorp).

### Ethics approval

The study was reviewed and approved by the Audit and Research Committee at HTD who granted ethical approval for the study and stated that individual patient consent was not required as this was a retrospective case note review.

## Results

### Patient characteristics

Demographic data are shown in Table 1. Most (n = 103, 83%). were transferred to UCLH from another centre. The median time from initial admission to transfer to UCLH was 16 hours (IQR 8–30 hours). The largest group were Caucasian (61, 49%), while 41 (33%) were African. Very few took antimalarial chemoprophylaxis. Only one patient claimed to be fully adherent with an appropriate regimen. Nearly all (116, 94%) acquired their infection in sub-Saharan Africa. Seven had a clear history of having received treatment for malaria in the past. Three were already known to have HIV infection. Median time since returning to the UK was 10 days (IQR 3–14). Median time from symptom onset to presentation was four days (IQR 3–6).

**Table 1:** Demographics

Male		78 (63%)
Age - years – median (IQR)		46 (35–55)
Ethnicity	Caucasian	61 (49%)
	African	41 (33%)
	Other	18 (15%)
	Unknown	4 (3%)
Previous malaria exposure	Raised outside endemic area	68 (55%)
	Born in endemic area + left > 2 years ago	46 (37%)
	Born and still resident in endemic area	7 (6%)
	Unknown	3 (2%)
Travel to sub-Saharan Africa Chemoprophylaxis	116 (94%)	
	None	87 (70%)
	Inadequate	25 (20%)
	Adequate	1(1%)
	Unknown	11 (9%)
Reported previous malaria		7 (6%)
Known HIV		3 (3%)
Time from returning to UK and admission - median days (IQR)		9.5 (3–14)
Time from symptom onset to seeking healthcare – median days (IQR)		4 (3–6)

Sixty-eight (55%) were classified as malaria-naïve. Forty-six (37%) were assumed to have “little” immunity to malaria as they had grown up in an endemic area. Seven were both born and usually resident in a malaria-endemic area at the time of admission and were assumed to have at least “some” degree of immunity.

### Malaria diagnosis

Median parasitaemia at admission, either to UCLH or the referring hospital, was 6.1% (IQR 2.6 - 18%). Ninety eight (79%) and 73 (59%) had a parasitaemia greater than 2% and 5% respectively. One hundred and eight (87%) and 82 (66%) patients had at least one parasite count greater than 2% and 5% respectively during admission. Twenty-two (18%) had schizonts at admission, and a further 53 (43%) had schizonts at some time during admission. Four had mixed infections, two with *P*. *malariae*, and one each with *P*. *vivax* and *P*. *ovale*.

### Management

One hundred and twenty three (99%) received intravenous quinine as their initial treatment, 93 (76%) of whom received a loading dose. Twenty were subsequently switched to an artemisinin derivative, 18 of whom received artesunate. One was treated with an artemisinin derivative from the start.

Thirty-three (27%), with a median parasitaemia of 25%, had an exchange transfusion performed. Forty six (37%) required ventilation at some time. Similarly 43 (35%) required renal replacement therapy and 43 (35%) required inotropic support. Median length of stay in ICU was 10 days (IQR 7–19) but several had a prolonged stay, the longest being 90 days.

### Complications of malaria

**Figure 1:**
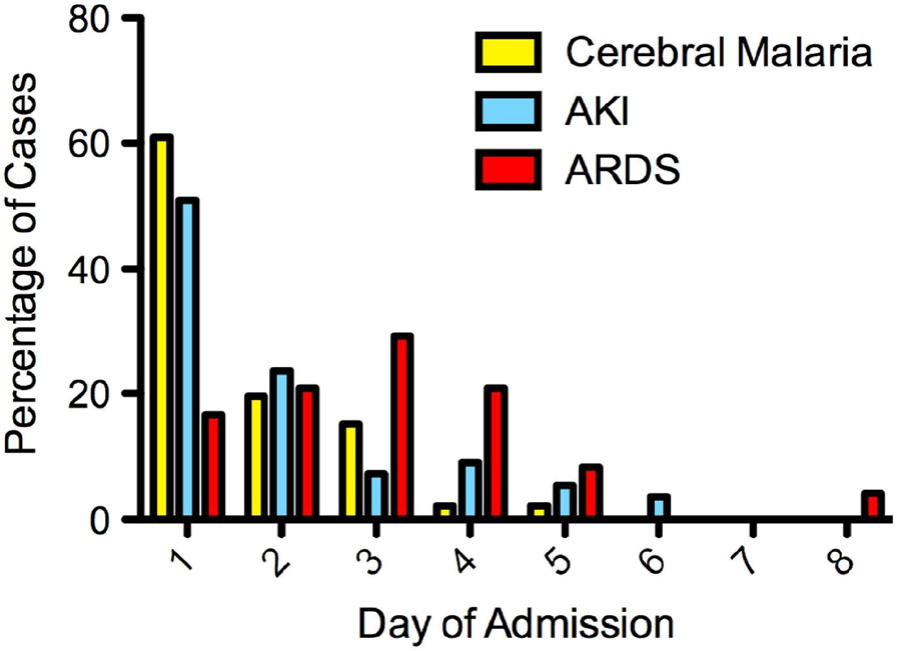
**Timing of major manifestations of severe malaria.** Cerebral malaria and acute renal failure most commonly occurred within 48 hours of presentation. ARDS was an uncommon manifestation of severe infection at presentation; most cases occurred from the third day of admission on.

**Figure 2:**
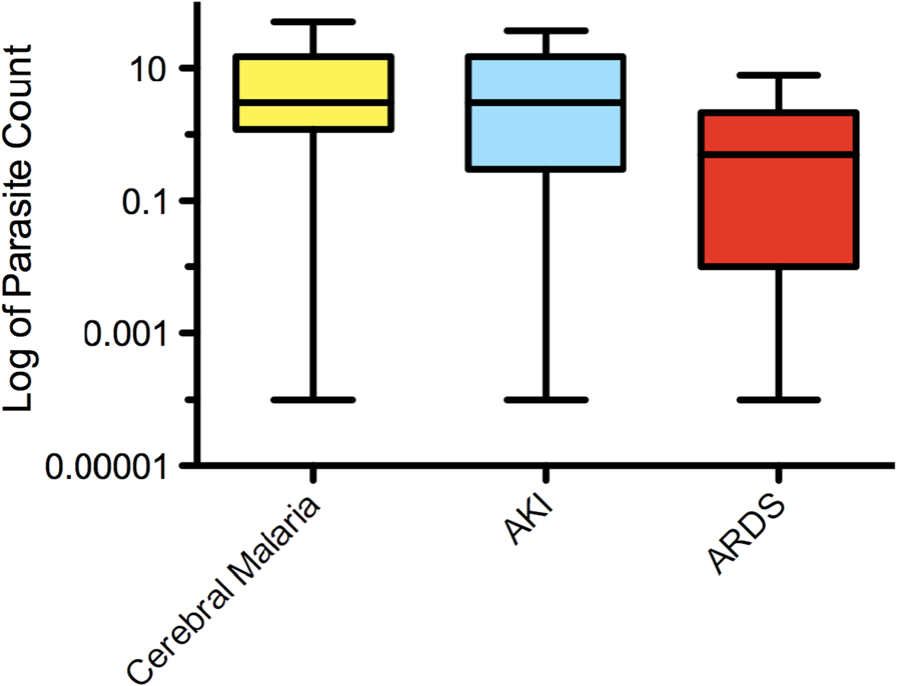
**Parasitaemia at the time of occurrence of major complications of malaria.** The median parasitaemia at the time when cerebral malaria and acute kidney injury developed was 3% compared to 0.5% when patients developed ARDS.

Patients met a median of three (IQR 2–5) WHO criteria for severe disease (Table 2). 80% of those with cerebral malaria and 75% of those with AKI developed these complications within 48 hours and tended to recover relatively quickly. In contrast, only two patients had ARDS at admission, while a further 22 developed ARDS later on, with a median time from admission to diagnosis of ARDS of three days (Figure 1). Patients with cerebral malaria and / or AKI had a higher parasitaemia at the time these complications became apparent compared to those patients who developed ARDS (Figure 2).

**Table 2:** Manifestations of severe disease

		**Present at admission**	**During admission**
Hyperparasitaemia	>2%	98 (79%)	108 (87%)
	>5%	73 (59%)	82 (66%)
Acidosis pH <7.35		37 (30%)	68 (55%)
AKI: creatinine >265 μmol/L		28 (23%)	55 (44%)
GCS <11		19 (15%)	44 (35%)
Shock: systolic BP <80 mmHg		15 (12%)	34 (27%)
Coagulopathy		7 (6%)	13 (11%)
Seizures		4 (3%)	9 (7%)
ARDS		2 (2%)	24 (19%)
Hypoglycaemia <2.2 mmol/L		0 (0%)	5 (4%)

More than half (68, 55%) were acidotic at some stage. Hypoglycaemia (defined as blood glucose <2.2 mmol/L) was not seen at admission but five patients, all treated with intravenous quinine, developed this at some point. Shock occurred in 34 (27%). Significant bleeding or coagulopathy was less common, occurring in 13 (11%) patients most of whom had laboratory evidence of DIC. One patient, who ultimately made a complete recovery, sustained spontaneous rupture of their spleen. Another developed purpura fulminans, which necessitated bilateral below-knee amputations.

### Co-infections

Six had a community-acquired co-infection; three had clinical and/or radiographic evidence of pneumonia while one had a coincidental buttock abscess. Two patients had a positive blood culture at admission, one *Staphylococcus aureus* and another *Klebsiella pneumoniae*. In one other, *Mycobacterium tuberculosis* was found in a sputum sample.

Twenty (16%) developed nosocomial infection during their stay most commonly caused by *Staphylococcus aureus* (n = 7), *Haemophilus influenzae* (n = 5) and *Pseudomonas* (n = 2). Sixteen developed pneumonia, two had proven fungal infection, one bacteraemia with *Staphylococcus aureus* and one an episode of *Clostridium difficile* colitis. Three patients, all African, were previously known to have HIV. Of 22 patients who were tested for HIV during admission, a further five were found to be positive, four of whom were African.

### Outcomes

One hundred and nineteen (96%) survived and all but one, the patient who developed purpura fulminans, ultimately made a complete recovery. Of the five patients who died, four were Caucasian; one was an African woman who had grown up in sub-Saharan Africa but left two years previously (Table 3). Their median parasitaemia was 18% (range 3.2 - 26%) and four had schizonts. All five were acidotic, all had AKI, three had either seizures or a GCS < 11 and two developed ARDS. Four required mechanical ventilation, four required renal replacement therapy, three had an exchange transfusion and in four of the five cases the malaria film was negative at the time of death. In only one could the cause of death be directly attributed to malaria, a patient who died of multi-organ failure on day three when their parasitaemia was still more than 2%. One patient died on day four as a result of cardiac arrhythmias which may have been precipitated by high-dose inotropes. The other three patients all died as a result of complications associated with prolonged ICU stay (disseminated fungal infection; multi-organ failure; staphylococcal pneumonia).

**Table 3:** Clinical characteristics of patients who died

**Patient No.**	**Demographics**	**Travel data**	**Manifestations of severe malaria**	**ICU support**	**Death**
1	31 year old female	Travelled to Zambia	Hyperparasitaemia	Exchange	Died Day 3
	Black African	No Chemoprophylaxis	Seizures	transfusion	Parasite Count 2.1%
	Raised in Endemic		ARDS	Ventilated	
	Region		AKI	RRT	
			Acidosis	Inotropes	
2	25 year old male	Travelled to Uganda and	Hyperparasitaemia	Exchange	Died Day 19
	White	Kenya	AKI	transfusion	Disseminated fungal infection
	Not-Raised in	No Chemoprophylaxis	Acidosis	RRT	
	Endemic Region	Treatment for malaria			
		started abroad: Chloroquine then Quinine			
3	83 year old male	Travelled to Kenya	AKI	Exchange	Died Day 9
	White	No Chemoprophylaxis	ARDS	transfusion	Multi-organ failure, shock with limb
	Not-Raised in		GCS < 11	Ventilated	
	Endemic Region		Acidosis	RRT	
			Shock	Inotropes	
			Coagulopathy		
4	41 year old male	Travelled to Gambia	Hyperparasitaemia	Ventilated	Died Day 4
	White	No Chemoprophylaxis	Acidodsis	Inotropes	VT followed by Cardiac Arrest
	Not-Raised in		AKI		
	Endemic Region		GCS < 11		
5	47 year old male	Living in Liberia for 2 Years	Hyperparasitaemia	Ventilated	Died Day 11
	White	No Chemoprophylaxis	AKI	RRT	Staphylococcal pneumonia
	Not-Raised in		Acidosis	Inotropes	
	Endemic Region				

Twelve had persisting renal impairment at the time of discharge from ICU, but all of these ultimately recovered. After discharge from hospital, three needed readmission, one patient developed an acute confusional state which was diagnosed as a post malaria neurological syndrome and the patient made a complete recovery while two had a relapse of *P*. *falciparum* which responded to a further course of treatment.

There was no demonstrable association between the amount of “immunity” to malaria (naïve, “little”) and the risk of severe ICU malaria (69% vs 63%, p = 0.54), cerebral malaria (67% vs 65%, p = 0.83), ARDS (78% vs 85%, p = 0.34) or AKI (61% vs 46%, p = 0.10). In logistic regression adjusting for the potential confounding factors outlined in the methods, no clinical factor was significantly associated with either death or severe ICU malaria.

A CAM score and an MSA score could be calculated at admission for 94 (76%) and 124 (100%) respectively. The positive predictive value of a CAM score <2 and an MSA score <5 for survival were 100% and 97% respectively. The positive predictive value of a CAM score ≥2 or an MSA ≥5 for death were 12% and 22% respectively. A CAM score ≥2 was significantly associated with an increased risk of death (12% vs 0%, p = 0.02) and of severe ICU malaria (50% vs 21%, p = 0.003). An MSA score ≥5 was associated with an increased risk of death (22% vs 3%, p = 0.04) but was not associated with a statistically significant increased risk of severe ICU malaria (44% vs 33%, p = 0.49).

## Discussion

This report shows that patients with severe malaria requiring admission to ICU who are managed at a specialist centre have a good outcome, with a mortality rate of only 4%. Consistent with previous reports, acute kidney injury, ARDS, and cerebral malaria were the most common complications. This report demonstrates that ARDS occurs later and at lower parasitaemia and emphasises the need for vigilance even after patients appear to be responding to treatment. The data reinforce the idea that there is a complex inter-relationship between ethnicity, “immunity” and the response to malaria. There were no significant differences between those individuals who had grown up in an endemic region and those patients who were malaria-naive. Co-infection with HIV was common, especially in patients who were African in origin. Finally, this report adds to the limited number of series reporting the efficacy and safety of parenteral artemisinins in severe malaria in a non-endemic setting [18-20]. The introduction of these agents has been associated with a dramatic reduction in the need for exchange transfusion.

In this series, the case-fatality rate was 4%. In contrast, a paper from Kilifi [21] showed that African children who had evidence of cerebral malaria, acidosis and severe anaemia had a 35% risk of death. In the SEAQUAMAT trial [22], among young Asian adults, the group treated with quinine had a mortality of 22%. In Africa, most deaths occur within the first 24 hours and children who survive beyond three days are much less likely to die. In this series, only one death occurred at a time when there was evidence of active malarial infection and the other four resulted from complications associated with intensive care, such as cardiac arrhythmia or nosocomial infection. Previous studies have reported a mortality rate between seven and 25% [5-11]. Recent data from the Malaria Reference Laboratory have shown a marked geographical variation in mortality from imported malaria from different parts of the UK [23]. It is possible that the lower mortality in various specialist centres may be a reflection of wider experience in the management of severe malaria.

Most patients were young, Caucasian travellers to Africa but the second largest group were Africans visiting friends and relatives. For both groups, adherence to chemoprophylaxis was poor. The UK Health Protection Agency have previously reported an increased risk of acquiring malaria in individuals travelling to visit friends and relatives [1]. Between 1994 and 2010, HTD treated 1451 patients for falciparum malaria who did not require admission to ICU. Among all these patients, Africans were significantly less likely than Caucasians to require ICU admission (OR: 0.26 95% CI: 0.18-0.38) which is in keeping with earlier reports [24]. However, among those who did require intensive care, Africans were no less sick. There were no differences in the number of WHO markers of severity between Africans and other ethnicities, suggesting that Africans admitted to ICU had no appreciable immunity to the disease. These findings highlight the inherent problems in using ethnicity as a proxy for “immunity” to malaria.

Cerebral involvement and/or AKI tended to be present on admission or develop within the first 48 hours. ARDS tended to develop later, often when there was little or no evidence of persisting malaria. Those caring for patients with severe malaria need to be aware that an initial response to treatment, manifest by a reduction in parasitaemia, does not necessarily mean a successful outcome.

Before the introduction of artesunate, this hospital recommended a six-unit exchange transfusion for patients with a parasitaemia greater than 20% or those with a parasitaemia greater than 10% but with evidence of end-organ dysfunction. In total, 33 (26%) patients had an exchange transfusion, including three of the five who died. Since switching to parenteral artemisinins, we have seen evidence of a rapid and dramatic decline in parasitaemia compared to the response to treatment with quinine. As a result, we have not performed an exchange transfusion since 2008 and, in agreement with other UK specialist centres such as the Liverpool School of Tropical Medicine (D Lalloo, personal communication), we no longer recommend this as a modality of treatment.

Children presenting to African hospitals with malaria often have positive cultures for gram-negative organisms, particularly non-typhoidal salmonellae. The rate of blood culture positivity can be as high as 12% [25,26]. Co-existing bacteraemia has been inconsistently reported in other series of imported malaria, with rates between 2.5 and 5% [5,6]; in this series, only two (1.6%) patients had positive blood cultures at admission, neither of which were salmonellae.

The extent of the interaction between HIV and falciparum malaria remains open to debate, but in sub-Saharan Africa, HIV positive pregnant women in particular appear to be at an increased risk of developing severe disease [27]. In this series, three patients were already known to be living with HIV/AIDS before they developed malaria; a further five were found to have HIV during admission, four of whom were African. A minimum of 17% of Africans therefore were positive for HIV, which will be an underestimate as not all were tested. These findings and the shared epidemiological risk factors for both diseases suggest that all patients presenting with severe malaria should be tested for HIV.

This study shows that both a CAM score <2 and an MSA <5 identified patients who would survive. However, these scores had limited ability to predict mortality and it remains unclear what role, if any, they may play in clinical practice in areas of the world where malaria is not endemic. No clinical factor was associated with a poor outcome but given the low case fatality rate, the study was under-powered to detect such a difference.

The main limitation of this study was its retrospective nature; as a result some data were missing or incomplete. Despite this, data capture was high, in particular data relating to ICU care, which were recorded systematically. Although the possibility of referral bias can not be excluded it is likely that our data are applicable to all patients with imported malaria requiring admission to ICU.

## Conclusions

This paper suggests that most adult patients with severe malaria who are managed in a specialist centre survive. Cerebral malaria and AKI tend to occur early in the disease, while ARDS develops later. Patients who die tend to do so because of complications associated with intensive care rather than malaria *per se*. Africans who have left an endemic area seem to be at the same risk of severe disease as malaria-naive individuals. The increasing use of artesunate has been associated with a reduced need for exchange transfusion, even in patients with very high parasitaemia. In contrast to children, bacterial co-infections were uncommon. No single factor consistently identified individuals at risk of death. Both the MSA and CAM scores accurately predicted survival in low-risk patients but had limited power to identify those at high risk of death. All patients with malaria should be screened for HIV.

## Competing interests

The authors declare that they have no competing interests.

## Authors’ contributions

MM extracted the data, carried out the analysis and wrote the initial draft of the paper. MA designed the research database and helped extract the data. MMS and PLC advised on the collection and analysis of data on the diagnosis of malaria and helped draft these sections of the manuscript. SB and GB generated the initial ICU dataset and advised on the collection and interpretation of all data relating to intensive care stay of patients. CJMW designed and carried out the statistical analysis. JD conceived of the study and helped draft the manuscript. All authors read and approved the final manuscript.

## Pre-publication history

The pre-publication history for this paper can be accessed here:

http://www.biomedcentral.com/1471-2334/13/118/prepub
